# The severity of psychosocial and functional morbidity among facially disfigured untreated noma cases in Ethiopia

**DOI:** 10.1186/s13104-023-06440-w

**Published:** 2023-08-07

**Authors:** Heron Gezahegn Gebretsadik

**Affiliations:** School of Global Health & Bioethics, Euclid University, Banjul, Gambia

**Keywords:** Noma, Morbidity, Functional, Psychosocial, Disfigurement, Face

## Abstract

**Objectives:**

Noma is a facially disfiguring disease that affects the oral cavity and midface structures. If left untreated, the disease is fatal. Noma causes severe cosmetic and functional defects in survivors, leading to psychiatric and social problems. However, there are limited data on psychosocial and functional sequelae associated with this disease. This cross-sectional study aimed to investigate psychosocial and functional morbidity among facially disfigured untreated Noma cases. Study participants were volunteer patients diagnosed with noma and awaiting surgery at two noma treatment centers in Ethiopia. A questionnaire derived from the APA’s DSM-5, the DAS59, and the Appearance Anxiety Inventory protocol was used to measure the psychosocial and functional morbidity of the cases between September 16 and October 10, 2022.

**Results:**

A total of 32 noma cases (19 women and 13 men) awaiting the next surgical campaigns were involved in the study. Study participants reported severe social (Likert score = 2.8) and psychological (Likert score = 3.0) morbidity. Functional limitation was moderate (Likert score = 2.9). This study has shown that psychosocial and functional morbidity in untreated noma cases in Ethiopia is substantial. Therefore, policymakers, clinicians, and researchers need to pay sufficient attention to providing adequate health care and preventing the occurrence of the disease in the long term.

**Supplementary Information:**

The online version contains supplementary material available at 10.1186/s13104-023-06440-w.

## Introduction

Noma is a progressive and fatal disease (85–90% death rate) that exclusively affects the poor. The disease is rare in developed countries but is still not uncommon in many developing countries, especially in the noma belt region, which stretches from the west (Senegal) to the east (Ethiopia) of Africa. Noma is a devastating orofacial gangrene that affects the orofacial anatomic region and leaves victims with short- and long-term functional, aesthetic, and related psychosocial impairments [1, 2]. Facial disfigurement has long been considered one of the most potentially distressing aspects of human anatomy, as the facial region is critical to self-concept, interpersonal relationships, and communication. A growing body of literature also notes that people with facial disfigurements have difficulty adjusting to their condition and experience social difficulties. Said that Noma-related abnormalities including skin induration, limited movement of the temporomandibular joints, and muscle involvement leading to progressive limitation of orofacial range of motion, results in overall psychosocial and functional (speech and mastication) morbidity among disease survivors [3, 4].

Although there is a growing body of literature on the psychosocial aspects of various facial disfigurements, little is known about Noma and its psychosocial impacts on the patient’s life. In this regard, only a few studies have shown that noma is associated with significant psychosocial and functional morbidity [5, 6]. This study was primarily initiated to investigate the psychosocial and functional morbidity among facially disfigured untreated Noma cases in Ethiopia. Filling the knowledge gap and providing sound scientific evidence to concerned bodies including policymakers, clinicians, and researchers were the other objectives of the study.

## Methods

A single-point cross-sectional study of noma patients awaiting facial reconstruction as part of the Facing Africa and Harar projects examined the disease’s psychosocial, functional, and aesthetic impact between September 16 and October 10, 2022. Volunteer patients who were 18 years old and above at the time of data collection and those with no history of Noma-related surgical intervention were considered for data analysis.

### Instruments

A questionnaire derived from the APA’s DSM-5 severity measure for social anxiety disorder (social phobia), the DAS59, and the Appearance Anxiety Inventory protocol was used to measure the psychological and social morbidity of the subjects. The questionnaire included 24 questions and contained demographic and clinical data. Of these, 10, 4, and 10 questions were designed to measure psychological, functional, and social outcomes, respectively. The questions were formulated based on Linker’s scales for measuring psychological and functional impairment, which were aligned with the core objectives of the study. 5- and 4-point Likert scales were used to scale the responses of study respondents.

A numerical analysis of the Linkert scale was performed to derive a numerical value (domain mood score) that quantifies and describes the cumulative response of the study participants in each domain (psychological, social, or functional) studied. Item sentiment scores were calculated for each question in the questionnaire before the corresponding domain sentiment scores were determined. Each item of the measure was scored on a 5-point scale (0 = never; 1 = occasionally; 2 = sometimes; 3 = most of the time; and 4 = all of the time). Similarly, item and domain scores (psychological and social) were also reduced to a 5-point scale, which allows the researcher to describe the severity of psychological and social morbidity among noma cases in terms of “none” (0), “mild” (1), “moderate” (2), “severe” (3), or “extreme” (4).

On the other hand, functional morbidity was assessed using Linker’s 4-point scale to measure the problem level. In this case, each item of the measurement scale was rated on a 4-point scale (1 - no problem at all, 2 - a minor problem, 3 - moderate problem, and 4 - severe problem). The same scales were used to describe item and functional area sentiment scores.

In addition, the item sentiment scores were calculated for each question of the questionnaire. First, the numerical value of each sentiment score was multiplied by the number of corresponding respondents for that item/question. Then, the sum of these scores was divided by the total number of study participants to calculate the sentiment score for each item. The same principle was used to calculate the sentiment scores for each domain.

The validity and reliability of the questionnaire were ascertained by conducting a pilot survey before applying it to collect the final data for analysis. The study participants were interviewed individually by the data collector (the principal researcher).

### Data analysis

The IBM SPSS (Statistical Package for Social Sciences) Statistics, version 29 version was used to analyze the collected data. Descriptive statistical analysis was applied to describe and present the findings of the study in simple and compact forms.

## Results

A total of 32 volunteer and accessible patients who were on the waiting list for future surgery under the Facing Africa and Harar projects between September 16 and October 10, 2022, were included in the study. Eighteen (56.3%) were female and 14 (43.7%) were male. The age of the study participants ranged from 20 to 61 years. After data analysis, it was found that the study participants reported severe social (Likert score = 2.8) and psychological (Likert score = 3.0) morbidity. Functional limitation was classified as moderate (Likert score = 2.9). Compared to males (9 out of 14), proportionally more females (15 out of 18) reported severe forms of social and psychological morbidity.

### Findings related to social morbidity (appearance anxiety)

As shown in Table 1, the study participants’ social morbidity was severe (Linkert score = 2.8). The detailed psychosocial morbidity associated with noma is described in the following table.

**Table 1:** Appearance Anxiety Inventory, Social morbidity test

Items/Questions (n = 10)	The 5-point Linkert scale/Sentiment levels with corresponding numerical values					Items/questions sentiment levels
	Not at all-0	Rarely-1	Sometimes-2	Often-3	All the time-4	
1. I often check my appearance (e.g., in mirrors, by touching with my fingers, or by taking photos of myself).	-	-	2 (6)	3 (26)	-	90/32 = 2.8, Severe
2. I compare aspects of my appearance to others.	-	1 (7)	2 (10)	3 (14)	4 (1)	73/32 = 2.3, Moderate
3. I avoid situations or people because of my appearance.	-	1 (8)	2 (2)	3 (18)	4 (4)	82/32 = 2.6, Severe
4. I think about how to camouflage or alter my appearance.	-	-	2 (4)	3 (17)	4 (11)	103/32 = 3.2, Severe
5. I avoid reflective surfaces, photos, or videos of myself.	-	-	2 (2)	3 (3)	4 (27)	126/32 = 3.9, Extreme
6. I try to camouflage (hide) or alter aspects of my appearance.	0 (4)	-	-	3 (2)	4 (26)	110/32 = 3.4, Severe
7. I brood (worry) about past events or reasons to explain why I look the way I do.	-	1 (1)	2 (3)	3 (3)	4 (25)	116/32 = 3.6, Extreme
8. I am focused on my appearance rather than my surroundings.	-	-	2 (6)	3 (21)	4 (5)	95/32 = 3.0, Severe
9. I discuss my appearance with others or question them about it.	0 (23)	1 (5)	2 (4)	-	-	13/32 = 0.4, None
10. I try to prevent people from seeing aspects of my appearance within particular situations (e.g., by changing my posture and avoiding bright lights).	-	1 (3)	2 (3)	3 (22)	4 (4)	91/32 = 2.8, Severe
**Domain sentiment level**	0 (23)	1 (24)	2 (40)	3 (126)	4 (103)	894/32*10 = 2.8, Severe

Table 1 shows the noma-related social morbidity in detail. The items and domain sentiment levels are shown with the calculated corresponding numerical values. The domain sentiment level value describes the overall social morbidity among the study participants.

### Findings related to psychological morbidity (social phobia)

Detailed results related to noma-induced psychological morbidity are described in Table 2. Study participants generally had severe psychological morbidity or social anxiety disorder (Linkert score = 3.0).

**Table 2:** Severity Measure for Social Anxiety Disorder (Social Phobia), Psychological morbidity test

Items/Questions (n = 10) Since the onset of the disease, I have…	The 5-point Linkert scale/Sentiment levels with corresponding numerical values					Items/questions sentiment levels
	Never-0	Occasionally-1	Half of the time-2	Most of the time-3	All the time-4	
1. felt moments of sudden terror, fear, or fright in social situations.	0 (2)	1 (5)	2 (3)	3 (15)	4 (7)	84/32 = 2.6, Severe
2. felt anxious, worried, or nervous about social situations.	0 (5)	1 (2)	2 (5)	3 (20)	-	72/32 = 2.3, Moderate
3. thought of being rejected, humiliated, embarrassed, ridiculed, or offending others.	0 (4)	-	-	3 (2)	4 (26)	110/32 = 3.4, Severe
4. felt a racing heart, sweaty, trouble breathing, faint, or shaky in social situations.	0 (3)	-	2 (2)	3 (23)	-	73/32 = 2.3, Moderate
5. felt tense muscles, felt on edge or restless, or had trouble relaxing in social situations.	0 (4)	1 (1)	-	3 (4)	4 (23)	105/32 = 3.3, Severe
6. avoided, or did not approach or enter, social situations.	0 (2)	-	-	3 (3)	4 (27)	117/32 = 3.7, Extreme
7. left social situations early or participated only minimally (e.g., said little, avoided eye contact).	0 (3)	1 (1)	-	-	4 (28)	113/32 = 3.5, Extreme
8. Spend a lot of time preparing what to say or how to act in social situations.	-	1 (3)	-	-	4 (29)	119/32 = 3.7, Extreme
9. distracted myself to avoid thinking about social situations.	0 (2)	-	2 (1)	3 (21)	4 (8)	97/32 = 3.0, Severe
10. needed help to cope with social situations (e.g., alcohol or medications, superstitious objects).	0 (6)	-	-	3 (26)	-	78/32 = 2.4, Moderate
**Domain sentiment level**	0 (31)	1 (12)	2 (11)	3 (114)	4 (148)	968/32*10 = 3.0, Severe

Table 2 illustrates the noma-associated psychological morbidity (social phobia). The domains’ items and sentiment levels are presented with the corresponding numerical values. The score for the sentiment domain shows the overall psychological morbidity of noma cases in Ethiopia.

### Findings related to functional morbidity

Results related to noma-induced functional morbidity are shown in Table 3. Noma-induced functional limitation was calculated to be moderate overall (linker score = 2.9) in those who reported at least one of the four functional impairments measured in the study.

**Table 3:** Functional morbidity test

Items/Questions (n = 4)	The 4-point Linkert scale/Sentiment levels with corresponding numerical values				Total number of positive responses	Items/questions sentiment levels
	Not at all a problem − 1 (Total number of negative responses)	Minor problem − 2	Moderate problem − 3	Severe problem − 4		
1. How do you rate your speaking problem?	1 (15)	2 (8)	3 (4)	4 (5)	17	48/17 = 2.8, Moderate problem
2. How do you rate your problem with opening your mouth?	1 (19)	2 (6)	3 (5)	4 (2)	13	35/13 = 2.7, Moderate problem
3. How do you rate your breathing problem?	1 (25)	2 (5)	3 (1)	4 (1)	7	17/7 = 2.4, Minor problem
4. How do you rate your mastication problem?	1 (11)	2 (7)	3 (5)	4 (9)	21	65/21 = 3.1, Moderate problem
**Domain sentiment level**	1 (70)	2 (26)	3 (15)	4 (17)	58	165/58 = 2.9, Moderate problem

Table 3 shows the noma-associated functional morbidity in detail. The domains’ items and sentiment levels are shown with the corresponding numerical values. The score for the sentiment level of the domain shows the overall functional morbidity of the study subjects.

## Discussion

With an estimated annual incidence of 140,000 cases worldwide, noma is a disease that poses enormous challenges to survivors due to its physical, psychological, and social impact [7]. Reports indicate that people with craniofacial disease are at known risk for several psychosocial problems, including low self-esteem, learning and language problems, depression, anxiety, and negative social reactions from peers [8–11]. When considering the psychosocial impact of noma, it is important to understand several elements of the disease [12]. The disfiguring facial changes caused by noma can directly impact mental and social health [13]. Similarly, the physical aspects of the disease, such as the altered ability to open the mouth, slurred speech, and excessive drooling, may affect social and psychological functioning as a secondary phenomenon by interfering with daily life [14–17]. According to studies, Noma is reportedly associated with long-term functional, aesthetic, and psychosocial impairments [16, 17]. Still, the impact of Noma on the psychosocial aspect of patients’ life is not well investigated. It is undeniable that certain psychological, social, and functional consequences of noma have been reported in the literature. However, these data are reported by few and mainly retrospective studies [6]. For example, noma survivors in Laos reported high levels of hopelessness and associated functional impairments [18]. Study participants who took part in this study also reported significant functional impairments (Likert score = 2.9). In Nigeria, a 37% psychiatric morbidity prevalence rate was found in noma cases with disfigured faces [14]. Similarly, the results of this study revealed severe noma-related social (Likert score = 2.8) and psychological morbidity (Likert score = 2.51).

In the present study, the functional, social, and psychological aspects of noma were found significant determinants of the study participants’ quality of life (QOL). The present study suggests that psychosocial morbidity is high in patients who are facially disfigured by Noma. The results also showed that the study participants were highly anxious because of their poor appearance and extremely dissatisfied with their facial expressions. Arguably, this dissatisfaction with facial expressions may lead to life-threatening behavioral problems. Given these risks, regular assessments of the psychosocial needs of people with noma must be emphasized. In addition, policymakers and experts need to be attentive in formulating a workable prevention strategy.

### Limitations

There was a language barrier between the investigator and two study participants, so a translator had to be consulted. The translator may not have translated the responses of these study participants correctly, as not everything that exists in a particular language community can be accurately translated into another language. This could have a slight impact on the results of the study.

### Electronic supplementary material

Below is the link to the electronic supplementary material.

Supplementary Material 1

## Data Availability

All data generated or analyzed during this study are included in this published article [and its supplementary information files].
