# Supplementary material for: The severity of psychosocial and functional morbidity among facially disfigured untreated noma cases in Ethiopia
Source: BMC Res Notes. 2023 Aug 7;16:162. doi: 10.1186/s13104-023-06440-w (PMC10408114; doi:10.1186/s13104-023-06440-w)

The APA DSM-5 Severity Measure for Social Anxiety Disorder (Social Phobia).


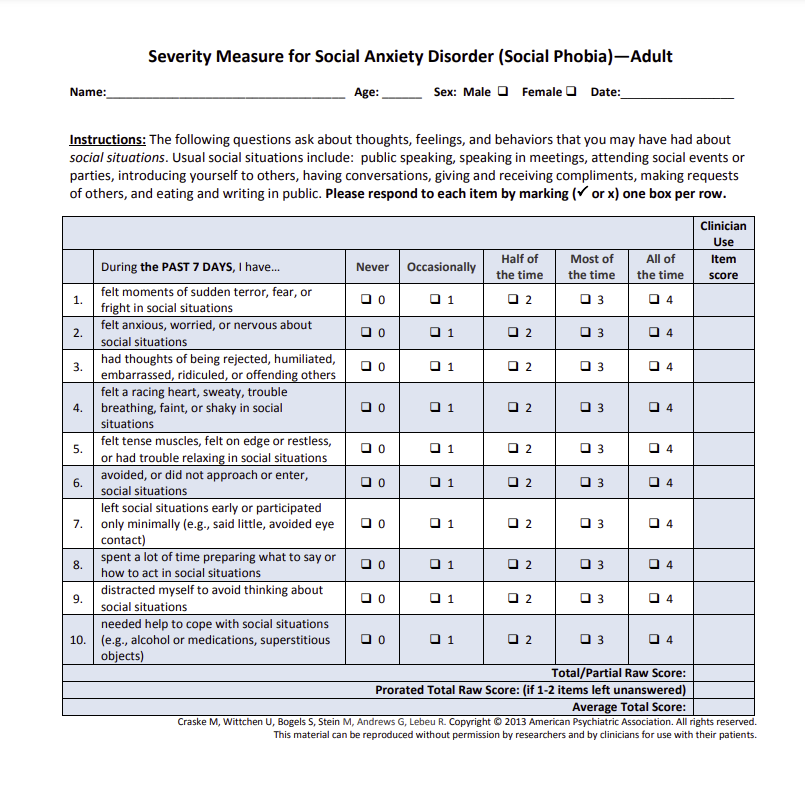


The Derriford Appearance Scale (DAS59).


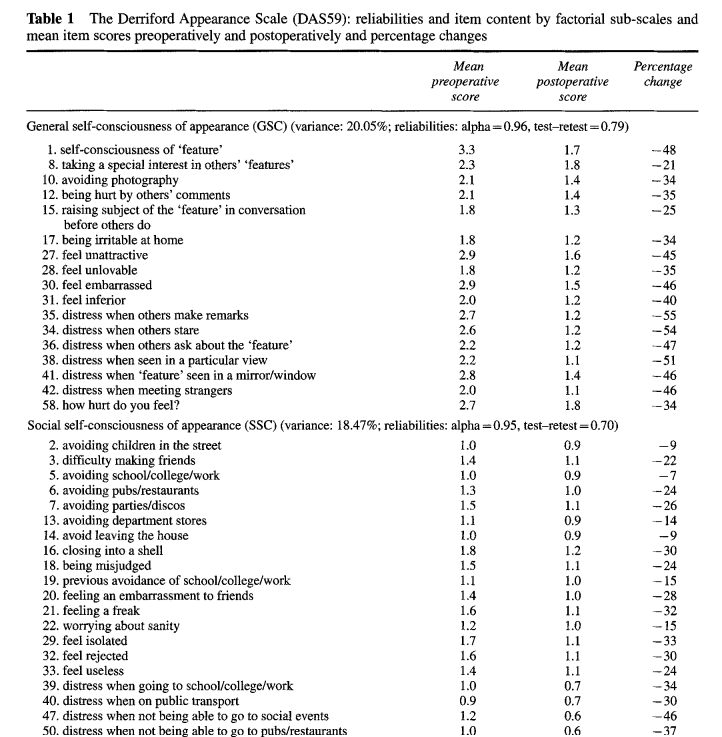


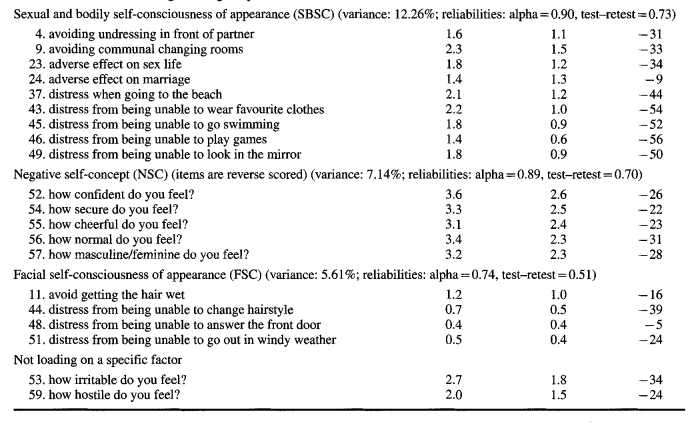


The Appearance Anxiety Inventory.


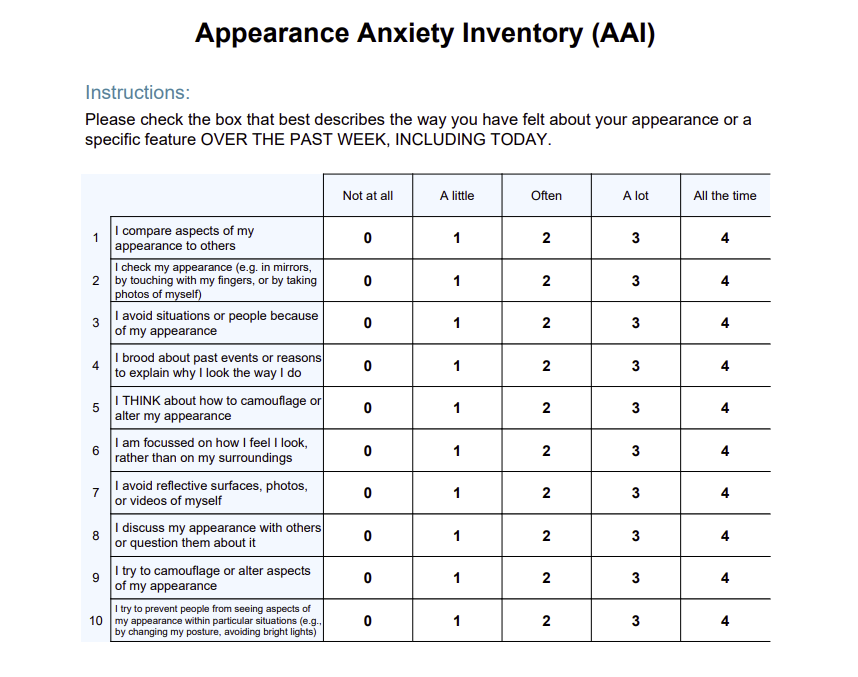

Supplement: Supplementary file 1 — Supplementary Material 1 [file 13104_2023_6440_MOESM1_ESM.docx]
